# An analysis of mobile genetic elements in three *Plasmodium *species and their potential impact on the nucleotide composition of the *P. falciparum *genome

**DOI:** 10.1186/1471-2164-7-282

**Published:** 2006-11-04

**Authors:** Pierre M Durand, Andries J Oelofse, Theresa L Coetzer

**Affiliations:** 1 Department of Molecular Medicine and Haematology, University of the Witwatersrand Medical School and National Health Laboratory Service, York Road, Parktown, 2193, South Africa; 2 Wits Bioinformatics, University Corner building, University of the Witwatersrand, Johannesburg, 2017, South Africa

## Abstract

**Background:**

The completed genome sequences of the malaria parasites *P. falciparum*, *P. y. yoelii *and *P. vivax *have revealed some unusual features. *P. falciparum *contains the most AT rich genome sequenced so far – over 90% in some regions. In comparison, *P. y. yoelii *is ~77% and *P. vivax *is ~55% AT rich. The evolutionary reasons for these findings are unknown. Mobile genetic elements have a considerable impact on genome evolution but a thorough investigation of these elements in *Plasmodium *has not been undertaken. We therefore performed a comprehensive genome analysis of these elements and their derivatives in the three *Plasmodium *species.

**Results:**

Whole genome analysis was performed using bioinformatic methods. Forty potential protein encoding sequences with features of transposable elements were identified in *P. vivax*, eight in *P. y. yoelii *and only six in *P. falciparum*. Further investigation of the six open reading frames in *P. falciparum *revealed that only one is potentially an active mobile genetic element. Most of the open reading frames identified in all three species are hypothetical proteins. Some represent annotated host proteins such as the putative telomerase reverse transcriptase genes in *P. y. yoelii *and *P. falciparum*. One of the *P. vivax *open reading frames identified in this study demonstrates similarity to telomerase reverse transcriptase and we conclude it to be the orthologue of this gene.

**Conclusion:**

There is a divergence in the frequencies of mobile genetic elements in the three *Plasmodium *species investigated. Despite the limitations of whole genome analytical methods, it is tempting to speculate that mobile genetic elements might have been a driving force behind the compositional bias of the *P. falciparum *genome.

## Background

Mobile genetic elements (MGEs) play a fundamental role as drivers of genome evolution, shaping both genes and genomes and often constitute a large fraction of the genome (for a review of mobile elements and genome evolution see [1,2]). The mutagenic effects of MGE behaviour are well documented and include a spectrum, from point mutations to whole genome restructuring. In addition, MGEs have occasionally become "domesticated" and evolved to fulfill essential functions in genome dynamics e.g. telomerase [2]. Consequently, MGEs and their derivatives have been identified in almost all organisms. Laboratory evidence has repeatedly demonstrated that MGEs can have either a beneficial [3] or detrimental [4] effect on the host's fitness depending on the downstream effects of transposition. To counteract the detrimental effects, some organisms have developed protective mechanisms against invading MGEs, such as the repeat-induced point (RIP) mutation system in *Neurospora crassa*, which silences repetitive elements by methylation and subsequent mutation of cytidine to thymidine nucleotides [5]. This explains the unusual situation in *N. crassa *where very few, if any, active MGEs have been detected.

**Figure 1:**
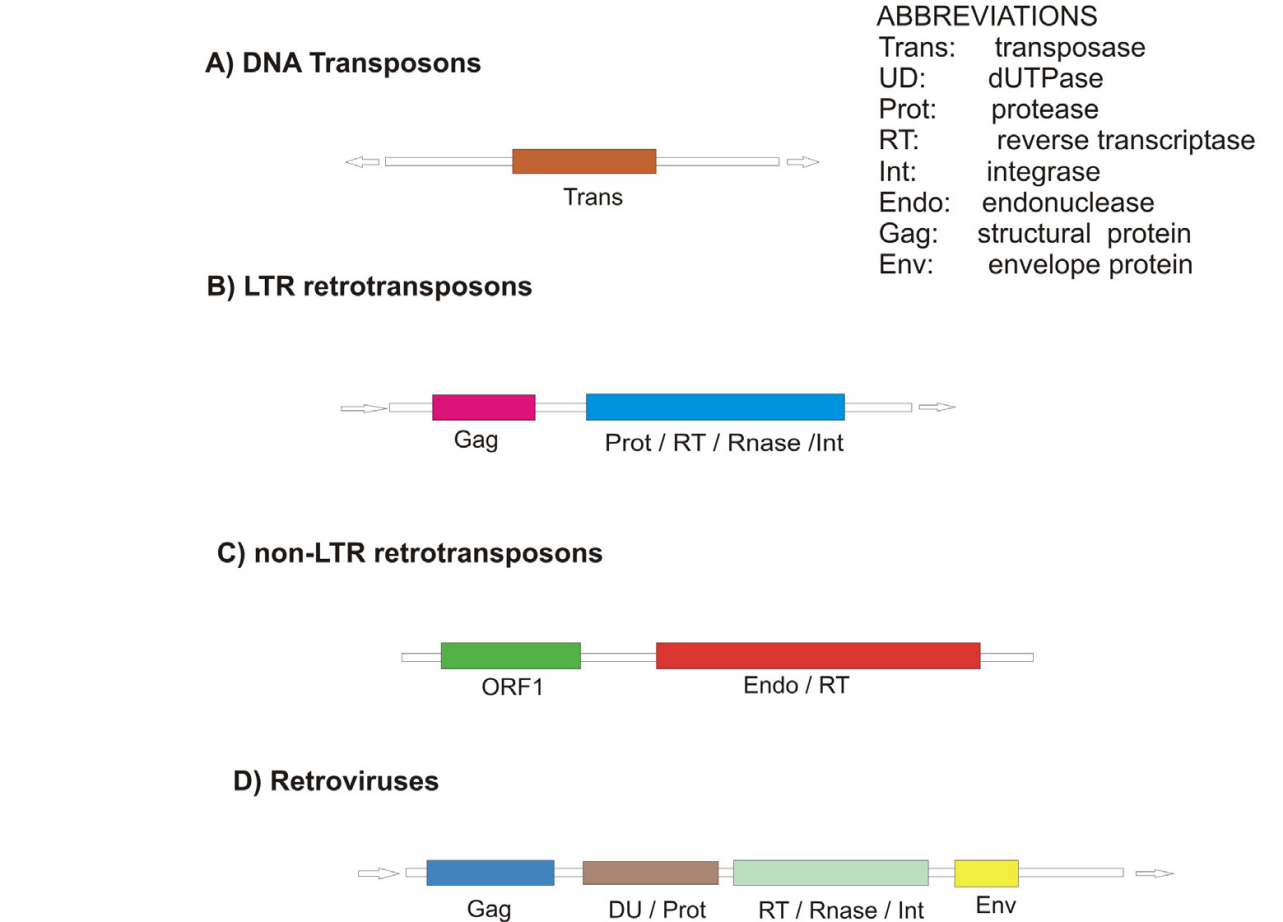
**Schematic representation of MGEs**. ORFs are represented by coloured blocks. Non-autonomous retrotransposons and mobile introns are not depicted here. Arrows represent flanking repeats.

MGEs are usually classified according to their mechanism of replication and whether they are autonomous (self-replicating) or non-autonomous (dependant on related elements for replication or mobility) [1]. The two broad groups are those which are mobile via either a DNA or RNA mechanism (Figure 1). The DNA group makes use of a self-encoded transposase that facilitates excision and integration from one site to another, while the RNA group utilizes an RNA intermediate and represents a more functionally and evolutionarily diverse group of elements. MGEs employing an RNA intermediate use reverse transcription to re-invade the genome, increasing their copy number along the way. In the RNA group, long terminal repeat (LTR) retrotransposons are evolutionarily related to retroviruses while the non-LTR retrotransposons form a discrete group and transpose via a unique target primed reverse transcription (TPRT) mechanism [6]. An offshoot of the non-LTR retrotransposons is the non-autonomous non-LTR retrotransposon group. These elements lack the enzymatic machinery for self-replication and mobilization and their survival depends on hijacking the replicative machinery of active non-LTR retrotransposons. MGE open reading frames (ORFs) code for the relevant enzymes that are required for replication and transposition (Figure 1).

Another intriguing group of mobile elements are the mobile introns. Divided into three groups based on their mechanisms of mobility (for a review of mobile introns see [7]), these are evolutionarily ancient MGEs and are believed to be the progenitors of non-LTR retrotransposons and eukaryotic spliceosomes. The group II introns have an extraordinary array of enzyme activity, including reverse transcriptase, maturase, RNase H and endonuclease activity as well as functional ribozymes. To date, group II introns have only been found in organellar genomes (chloroplast and mitochondrial) and a few cyanobacteria.

Very little is known about the occurrence of MGEs in *Plasmodium *species and the role they may have played in the evolution of the parasites' genomes. Initial reports after sequencing of the *P. falciparum *and *P. y. yoelii *genomes suggested that there may be a complete absence of MGEs in *P. falciparum *and no comment was made about MGEs in *P. y. yoelii *[8,9]. There is bioinformatic and laboratory evidence for the presence of a domesticated derivative of a non-LTR retrotransposon, namely telomerase, in *P. falciparum *and *P. y. yoelii *[10-12]. The only other evidence we could find in the literature for MGE-derived or related sequences in *Plasmodium *spp. are the Alu elements (non autonomous non-LTR retrotransposons) located within the antigen coding genes of *P. vivax *[13] and the TAREs (telomere associated repetitive elements) found within the multigene superfamilies that code for antigen variation e.g.*var*, *rif *and *stevor *in *P. falciparum *[14]. The function of these repeats is uncertain although it is proposed that they facilitate antigenic change and immune evasion.

We report here a comprehensive genome analysis of potential MGEs and their derivatives in *P. falciparum*, *P. y. yoelii *and *P. vivax*. This provides further insight into *Plasmodium *genome evolution with particular reference to the compositional bias in *P. falciparum*.

## Results

### Potential MGE domains determined from InterPro and Pfam databases

Table 1 lists all the *P. falciparum *and *P. y. yoelii *entries with MGE domains in InterPro [15] and Pfam [16] databases. Seven *P. y. yoelii *and four *P. falciparum *ORFs were identified. The four *P. falciparum *ORFs include one with similarity to a phage integrase, one with similarity to an intron-encoded nuclease and two domesticated derivatives. InterPro (and Pfam) domains that did not yield any hits in the *Plasmodium *database PlasmoDB [17] were:

IPR000477 (PF00078) Reverse transcriptase; IPR001584 (PF0065) Integrase catalytic domain; IPR002156 (PF00075) Ribonuclease H fold; IPR001037 (PF00552) Retroviral integrase C terminal; IPR003308 (PF02022) Integrase N terminal Zn binding domain; IPR002050 (PF00429) ENV polyprotein; IPR004875 (PF03184) Endonuclease CENP-B protein; IPR008906 (PF05699) hAT element; IPR003322 (PF02337) retroviral GAG protein; IPR005162 (PF03732) retrotransposon GAG protein; IPR000442 (PF01348) Group II intron-encoded maturase; IPR002492 (PF01498) DNA-binding part of catalytic domain of Tc3 transposase; IPR008042 (PF05380) Aspartic protease from PAO retrotransposon family; IPR000305 (PF01541) Group I excinuclease; IPR003545 Telomerase reverse transcriptase; IPR001162 Excinuclease; IPR006350 Intron endonuclease group I; IPR008180 (PF00692) DeoxyUTP pyrophosphatase; IPR001995 (PF00077) Peptidase A2A, retrovirus; IPR000721 (PF00607) retroviral nucleocapsid protein gag (p24).

### HMM for group II introns

There were no hits identified in the *P. falciparum *organellar genomes (ORFs>50 amino acids) in PlasmoDB with E values less than 100 that demonstrated significant similarity to either of the two group II intron HMMs. Where significant similarity did arise, this was due to upstream exons being fused to the mitochondrial group II introns used in generating the HMM, e.g. PFB0795w is the *Plasmodium *gene orthologue of the ATP synthase gene and corresponds to a fused exon located upstream of several mitochondrial group II introns.

### Reverse transcriptase domain

**Figure 2:**
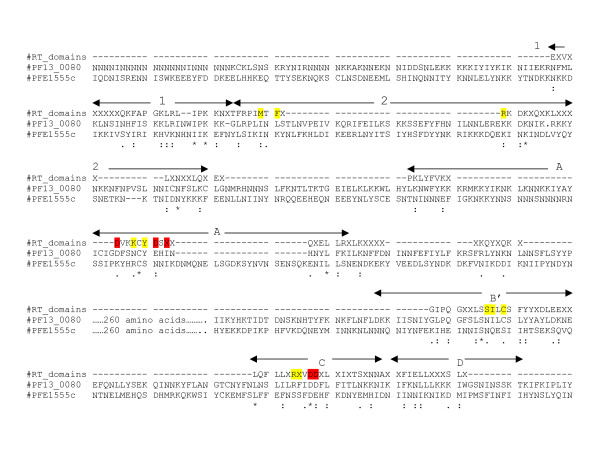
**Multiple sequence alignment of conserved RT domains and two potential RT-encoding genes in *P. falciparum***. #RT_domains is the RT consensus sequence developed in this study. It includes the fingers (1 and 2) and palm (A, B', C and D) domains. #PF13_0080 is the putative PfTERT identified by Figueiredo *et al*, 2005 [12]. #PFE1555c is a hypothetical protein with similarity to the RT consensus sequence developed in this study. The alignment was performed using ClustalW with default parameters. Residues shaded in yellow have been demonstrated to be in contact with nucleic acid [29]; residues shaded in red are critical for RT activity [30]. * residues common to all threesequences. : conserved residues. . semi-conserved residues

The WashU-BLASTP 2.0 search of PlasmoDB using the RT consensus sequence developed in this study, detected two *P. y. yoelii *(PY00479 and PY03683) and four *P. falciparum *(PF13_0080, PFC0960c, PFE1555c and PFC0165w) ORFs with similar E values (less than 10). Of the two *P. y. yoelii *genes, PY00479, which is annotated as a putative telomerase reverse transcriptase in PlasmoDB, was not detected by the InterPro TERT HMM. PY03683 is a hypothetical protein of 48 amino acids, making it too short for any meaningful comparison, and was not investigated further. Of the four *P. falciparum *genes only PF13_0080 and PFE1555c appear to have significant non-random homology in the most conserved residues, despite the E values of all four being similar. PF13_0080 is annotated as a putative telomerase reverse transcriptase in PlasmoDB and bioinformatic evidence for this has already been published [11,12]. PFE1555c is a hypothetical protein. An alignment of the RT consensus sequence generated in this study with these two genes is demonstrated in Figure 2. Some of the highly conserved residues, as well as some residues shown to be essential for RT activity, are present in both sequences.

**Figure 3:**
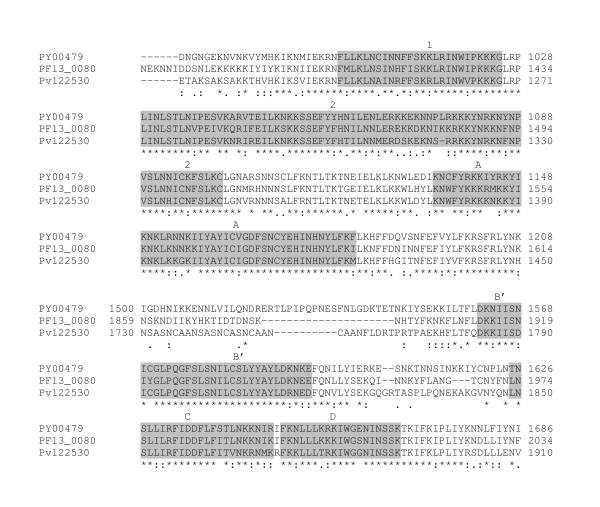
**Multiple sequence alignment of PyTERT, PfTERT and PvTERT**. Evidence for the putative *P. y. yoelii *(PY00479) and *P. falciparum *(PF13_0080) TERTs has been published previously [10-12]. The candidate *P. vivax *TERT (Pv122530) was identified in this study. Shaded blocks represent RT conserved domains. * residues common toall three sequences. : conserved residues. . semi-conserved residues

The RT consensus sequence was also used to probe the *P. vivax *genome available from TIGR [18]. A WashU-BLASTP (E value = 1, Blosum35 matrix) search revealed one hit that demonstrated significant similarity to the RT domain, including many of the highly conserved residues. This ORF (Pv122530) exhibits significant sequence similarity to the putative TERTs in *P. falciparum *and *P. y. yoelii*. Figure 3 is an alignment of these two sequences with the putative *P. vivax *TERT (PvTERT) which is reported here for the first time.

An orthologue of the PFE1555c gene, Pv079710, was identified by a simple BLASTP search of the *P. vivax *genome.

### MGE domains in the *P. vivax *genome

Ten *P. vivax *ORFs were identified by a BLASTP homology search as orthologues of the *P. y. yoelii *and *P. falciparum *genes located in InterPro (without a corresponding match in Pfam). HMMER searches of the *P. vivax *genome using Pfam_fs HMMs identified 28 ORFs containing MGE domains. In total therefore, 40 *P. vivax *ORFs containing MGE domains were identified and these are listed in Table 2.

## Discussion

We report here the presence of MGE signature domains in potential protein coding sequences of three *Plasmodium *species: six in *P. falciparum*, eight in *P. y. yoelii *and 40 in *P. vivax*. In each species there are three potential domesticated derivatives of MGEs. They are meiotic recombinase, DNA repair protein rad51 and TERT. Although these three proteins are common to all eukaryotes and are no longer active MGEs, they have clearly defined MGE domains and are included for completeness. Recently, identification of the putative TERT genes in *P. falciparum *and *P. y. yoelii *has been of interest and bioinformatic evidence of this has been published [11,12]. We have identified a candidate for the TERT orthologue (PvTERT) in *P. vivax *using the conserved RT sequence developed in this study (Figure 3). We cannot discount that other, as yet uncharacterized, domesticated derivatives may be among the ORFs detected.

Of the remaining ORFs identified, only three are present in *P. falciparum*. One is homologous to the catalytic domain of a phage integrase, a second to an intron-encoded nuclease and a third to a reverse transcriptase. Although the latter does not contain all the highly conserved residues, there is significant homology to the RT consensus sequence used in this study and warrants further investigation (see Figure 2). The highly conserved "DD" motif, located in domain C, aligns with a "DE" residue pair in PFE1555c. While almost all RT sequences have been found to contain the "DD" motif, some non-LTR retrotransposons used in InterPro to generate the RT HMM (IPR000477) have a glutamic acid (E) substitution for aspartic acid (D).

There were seven ORFs identified in *P. y. yoelii *with similarity to signature domains of MGEs: two transposases, two phage integrases, one intron-encoded nuclease and two domesticated derivatives. One of the ORFs with similarity to a phage integrase domain was a putative *yir*3 protein (PY05943). The *yir *family in *P. y. yoelii *is analogous to the *rif, stevor *and *var *superfamilies in *P. falciparum *where recombination events play an important role in antigen switching. Analysis of the *P. vivax *genome yielded a relatively large number of RT domains, 23 in total. The reasons for this are not clear and require further investigation.

### Whole genome analytical methods

The InterPro entries for TERT failed to detect the two TERT orthologues in *P. falciparum *and *P. y. yoelii*, probably due to the stringency of the HMM search. This highlights the pitfalls of using generic bioinformatic programmes for studying a genome as exceptional as that of *Plasmodium*. The problems associated with using bioinformatic methods to compare divergent sequences have been discussed before [19]. It must be stressed therefore that the findings presented here cannot be extrapolated further than the limits of the methods used. In particular, MGE detection in compositionally biased genomes may be affected by the lack of sensitivity of the scanning techniques employed. It is also possible that not all MGEs have been described to date and therefore not detected in this comparative study. We did, however, refine the genome mining process in two ways. Firstly, we employed biological data (crystal structure and point mutation data for RT sequences) to generate a consensus RT sequence to expand the scanning process. This increased the sensitivity and detected the *P. y. yoelii *and *P. falciparum *TERTs and the PFE1555c ORF discussed above. Secondly, less stringent statistics were used in the scanning process. While it is more common to use E values of 10^-5^, we used values of 1 or 10. Despite the relatively high E values there were few positive hits (see Results) and we eliminated potential false positives by excluding sequences that demonstrated random homology patterns or which demonstrated no homology in the conserved domains.

To further refine and validate our data we excluded ORFs that could potentially represent contaminating DNA. This was done by scanning the genomes of the vertebrate hosts that were used in the respective sequencing projects. All available *Saimiri boliviensis boliviensis *sequences and the related *Mucaca mulatta *genome were searched for ORFs with similarity to the potential *P. vivax *MGEs identified in this study. Similarly, the *Mus musculus *and the *Homo sapiens *genomes were scanned for ORFs with similarity to the potential MGE domains detected in *P. y. yoelii *and *P. falciparum *respectively (see Methods). No potential contaminating vertebrate host DNA was identified amongst the potential *P. vivax *and *P. falciparum *MGE domains. However, several *P. y. yoelii *sequences demonstrated >80% identity to ORFs in the *M. musculus *genome and were rejected from the data as being contaminating sequences. These were PY06984, PY07613, PY07841, PY07669, PY07288, PY06363 and PY07014. The fact that these ORFs have homologues in other rodents e.g. *Rattus norvegicus*, confirms they are contaminating sequences.

### MGEs and the *P. falciparum *genome

Given the above limitations and that the evidence presented here would need to be confirmed experimentally, these findings raise some interesting questions. While there are potentially several MGEs of different classes in *P. y. yoelii *and *P. vivax*, *P. falciparum *appears to contain very few. MGEs are ubiquitous in nature and are considered important catalysts for genome evolution. An interesting exception is the fungal genus *Neurospora*, which has the widest array of genome defence mechanisms found in eukaryotes, including the repeat-induced point mutation (RIP) mechanism whereby cytidine is mutated to thymidine [20,21]. This has had a profound impact on the organism's genome: it has extinguished active MGEs and driven the genome to become AT-rich, particularly in the non-coding regions [22]. Since the discovery of RIP-induced MGE silencing in *Neurospora*, this mechanism has been demonstrated in all kingdoms of life. Most recently, Kuhlmann *et al *have demonstrated that a RIP-like mechanism is capable of silencing retrotransposons in *Dictyostelium *[23]. This not only extinguishes MGE activity, but drives the AT bias in repeated sequences by subsequent deamination of methylated cytidine residues. These two features – an AT-rich genome (increasing to ~90% in non-coding regions) and an apparent lack of active MGEs – are notable features of *P. falciparum*. In addition, two putative DNA methyltransferases (PF11_0284 and PF13_0286) and a putative cytidine deaminase (PF13_0259) have been identified in *P. falciparum*, suggesting that the potential exists for methylation and deamination of cytidine residues in the genome.

The evolutionary forces that drive genomes to become compositionally biased are complex and largely theoretical. The arguments put forward depend upon the ecological niche of the individual organism and proposed hypotheses are therefore species specific. No hypothesis has been forthcoming for the AT richness found in *P. falciparum*. Since the initial effect of MGE invasion on the host genome is almost always negative [4], a selective pressure would exist to minimize MGE activity. Based on the data presented in this study, we propose the following two hypotheses, both of which may offer an evolutionary explanation for part of the AT richness of the *P. falciparum *genome:

i) A genome protective mechanism, such as the RIP-induced silencing of transposable elements, has been a potential driving force behind the compositional bias of the *P. falciparum *genome.

ii) The AT-rich genome of *P. falciparum *has been selected for as a result of its inherent resistance to MGE invasion.

The finding that the putative RIP machinery is represented in the *P. falciparum *genome lends support to the first hypothesis. The second hypothesis is based on findings that various MGEs may require specific recognition sequences as potential sites for integration e.g. the *mariner *element from *Drosophila mauritiana *appears to recognize specific nucleotide patterns as potential sites for integration [24].

## Conclusion

A comprehensive genome analysis of the three complete sequences of *P. falciparum*, *P. y. yoelii *and *P. vivax *revealed that a decrease in the frequency of transposable elements coincides with an increase in AT bias. A causal relationship between these two parameters remains to be established since there are limitations in the currently available bioinformatic screening methods. However, the hypothesis that a genoprotective mechanism exists in *P. falciparum *and that this may contribute to the compositional bias should be considered. Whole genome analytical methods that are particularly suited to compositionally biased genomes would be of value in further understanding the *P. falciparum *genome.

## Methods

### Protein databases

Protein sequences, as opposed to nucleic acid sequences, were used as they tend to be more sensitive and specific and the signature domains used in this study are all protein coding. Protein domain signatures characteristic of MGEs and their derivatives were identified based on their annotation in InterPro (release 12) [15] and Pfam-A (version 19) [16] databases and for which supportive evidence could be found in the literature. MGE signature domains that are present in host proteins were considered potential domesticated derivatives and were included in this study e.g. telomerase is a domesticated derivative of non-LTR retrotransposons and contains the signature domain of a reverse transcriptase (RT) [25]. The keywords used covered all potential known domains in MGEs as well as different classes of MGEs. InterPro and Pfam entries that did not yield any relationship to known MGEs or to domesticated were excluded. The keywords used were as follows:

(i) for signature domains : transposase, reverse transcriptase, ribonuclease, integrase, protease, endonuclease, zinc finger, GAG polyprotein, maturase and recombinase.

(ii) for classes of MGEs: transposon, retroelement, retrovirus, non-LTR retrotransposon, LTR retrotransposon and group II introns.

### HMM for group II introns

The signature domains of group II introns are not well represented in InterPro or Pfam. Hidden Markov models (HMMs) were therefore generated for mitochondrial and chloroplast forms of this MGE (see [26] for original development of HMMs and [27] for a review of HMMs). The full lengths of individual group II introns were used to generate models as some of the signature domains (eg. domain X) were too short to generate any meaningful results. The 14 chloroplast and 19 mitochondrial group II introns used to build HMMs were as follows (accession numbers are those in GenBank):

Chloroplast: X04826 (trnK I1), Z00044 (trnK I1), Z11741 (trnK I1), JN0302 (trnK I1), X52765 (trnK I1), X15901 (trnK I1), X57097 (trnK I1), D11467 (trnK I1), X04465 (trnK I1), P19593 (petD I1), X55877 (rbcL I1), X70810 (psbC I2, psbC I4 and psbD I8).

Mitochondrial: X57968 (nad1 I4), M63034 (nad1 I4), M30176 (nad1 I4), M68929 (SrDNA I1, atp9 I1, atpA I1, atpA I2, cob I3, coxI I1, coxI I2, coxII I2), X14669 (coI I1), X55026 (COI I1, COI I4, ND5 I4), M62622 (cox1 I1, cox1 I2), X57546 (cox1 I1), X54421 (cob I1).

The software package HMMER 2.3.2 [28] was used with default settings to generate HMMs. Only the *P. falciparum *organellar genomes were investigated.

### Reverse transcriptase domain

The RT domain is common to all MGEs (including group II introns), except DNA transposons, and has been the most intensively studied MGE domain. However, the divergent and abundant nature of RT containing sequences makes it difficult to construct a meaningful HMM or consensus sequence for use in BLAST searches and alignments. Based on biochemical knowledge of the RT domain such as its crystal structure and point mutation data [29,30], we generated a RT consensus sequence. This complimented the detection of potential MGEs and these findings were validated by comparison with the results from the InterPro and Pfam databases. Six of the seven conserved regions common to most RTs were included, namely domains 1 and 2 (fingers) and domains A, B', C and D (palm). Domain E (thumb) was not included as it was not possible to generate a meaningful sequence using this method (for a review of RT domains in MGEs see [31]).

An initial consensus sequence of the RT domain was generated that was representative of RT-encoding MGE classes (retroviruses/LTR retrotransposons, non-LTR retrotransposons, and group II introns) and two related domesticated MGEs (a telomerase and a telomere associated non-LTR retrotransposon). The consensus sequence was generated from six sequences, all of which had laboratory evidence of RT activity *in vitro*, using the EMBOSS:cons software from the European Bioinformatics Institute [32] with the default scoring matrix. The RT regions of the following six sequences were used: mitochondrial group II intron cobI1 (X54421), chloroplast group II intron psbA (AY290861), HIV1-RT (AAY66384), *T. thermophila *telomerase reverse transcriptase TtTERT (AF062652), LINE element DONG (L08889) and *D. melanogaster *telomere associated retrotransposon DmTART (U14101). Accession numbers are those in GenBank. This initial consensus sequence was refined manually in the following way:

i) Residues that were determined by crystal structure of a retrovirus RT to have sidechain or mainchain contact to either nucleic acid, dNTPs or Mg^2+ ^cofactor [29] and that were present in at least one of the six retroelements above were inserted in the consensus sequence.

ii) Amino acid residues that have been demonstrated to be essential for RT activity *in vitro *[30] were inserted in the consensus sequence.

iii) A ClustalW alignment [33] (software available at [32]) with default parameters of the six sequences used to generate the consensus sequence was analysed. Where the same residue was present more than once in the same position in the alignment, it was considered significant due to the diversity of the alignment and was included in the consensus. If there was more than one residue represented more than once, both residues were included as alternatives at that position.

Table 3 shows the final RT consensus sequence used in the analysis.

### Genome mining

MGE domains in InterPro and Pfam were investigated for *P. falciparum *and *P. y. yoelii *entries. The chloroplast and mitochondrial group II intron HMMs were used to search the *P. falciparum *organellar genome in PlasmoDB release 4.4 using the software package HMMER 2.3.2.

Pfam_fs HMMs were extracted from the database and used to search the *P. vivax *genome in TIGR. An E value of 1 and the individual trusted cutoff value for each Pfam entry were used in mining the *P. vivax *genome so that results could be compared. Since not all InterPro entries have a corresponding Pfam entry, *P. falciparum *and *P. y. yoelii *sequences located only in InterPro were used to search for orthologues in *P. vivax *using WashU-BLASTP at TIGR.

*P. falciparum *was cultured in human blood and *P. y. yoelii *and *P. vivax *were cultured in their respective host organisms in the sequencing projects [8,9,34]. MGE signature domains that represented contaminating vertebrate host DNA were therefore excluded from this study. For *P. y. yoelii *this was done by a BLASTP search of the *M. musculus *genome. The complete genome of *S. boliviensis boliviensis *(*P. vivax *host) has not been sequenced. We therefore screened the available *S. b. boliviensis *sequences as well as the complete genome of a related primate, *Mucaca mulatta*, for any of the *P. vivax *MGE domains identified. The *P. falciparum *MGE domains were screened against the human genome. The genomes of *M. musculus*, *M. mulatta *and *H. sapiens *are available at [35].

## Authors' contributions

PD conceptualized and designed the study, carried out the analysis and drafted the manuscript. AO assisted with and critically reviewed the bioinformatic methods. TC conceptualized the inclusion of *P. vivax *in the study, helped draft the manuscript and gave considerable intellectual input in analyzing the results. All authors read and approved the final manuscript.

## Figures and Tables

**Table 1:** InterPro and Pfam mobile genetic element signature domains identified in *P. falciparum *and *P. y. yoelii*.

**InterPro # (Pfam #)**	**InterPro Description**	***Plasmodium *ORF**
IPR004244 (PF02994)	Transposase_22/L1 element	PY07579 (PORF1)
IPR002104 (PF00589)	Phage integrase catalytic domain	MAL13P1.42 (hypothetical) PY04273 (hypothetical)
IPR010998	Lambda phage integrase-like domain	PY05943 (yir3 protein, putative), MAL13P1.42 (hypothetical), PY04273 (hypothetical)
IPR001207 (PF00872)	Transposase, mutator type	PY07767 (transposase, mutator type)
IPR003611 (PF07460)	Intron-encoded nuclease 2	PFC0925w (hypothetical), PY00434 (hypothetical)
IPR011940	Meiotic recombinase	PY05593 (DNA repair protein rhp51), MAL8P1.76 (meiotic recombination protein dmc1-like)
IPR011941	DNA repair protein rad51	PY03786 (rad51 homologue), PF11_0085 (rad51 homologue)

Seven *P. y. yoelii *and four *P. falciparum *ORFs were identified. The ORFs MAL13P1.42 and PY04273 were duplicated by the two InterPro domains IPR002104 and IPR010998.

**Table 2:** *P. vivax *ORFs containing MGE domains.

**Pfam entry**	**Number of ORFs**	**E values and/or sequence alignment scores**
PF03184 Endonuclease CENP-B protein	2	E values -9 and -10 Scores 37 and 39
PF00692 DeoxyUTP pyrophosphatase	1	E value -8 Score 35.5
PF00078 Reverse transcriptase	23	E values -8 to -27 Scores 35 to 104
PF00589 phage integrase catalytic domain	1	E value -13 Score 52
PF07460 Intron-encoded nuclease 2	1	E value -3 Score 21

**Similarity to RT sequence developed in this study**		
Determined by BLASTP search	2	E values -2 and -3

**Similarity to *P. y. yoelii *and *P. falciparum *ORFs not in Pfam**		
Determined by BLASTP search	10	E values -4 to -168

ORFs containing MGE domains were identified in the genome in three ways: i) a HMMER search using Pfam_fs HMMs ii) a BLASTP search using the RT consensus sequence developed in this study, and iii) a BLASTP search using the *P. y. yoelii *and *P. falciparum *MGE domain sequences identified from InterPro but not located in Pfam. Only the Pfam entries with significant hits are listed.

**Table 3:** The six conserved domains used in this study to represent the RT sequence of RT-encoding MGEs.

**RT region**	**Domain**	**Sequence**
Fingers	1	II^1^XXL^1^[KG]S[GY]P^1^E^1^N[FP]K^1^XG^1[^RP][IV]XXI^2,3^[PL]K^2,3^[PK]D^1^[GK][GS]
Fingers	2	XR^2,3^P^2^[LI]^2,3^[TS]XXS^3^XXXK^2,3^IXQ^1^XV[IL]X[IK]XL^2^XXXXEXK
Palm	A	K^1^XXX[WS]X[TV]XL^1^D^2,4^I^1^K^2^K^1,3^CF^2,3^D^2^S^2^I^2^P^1^H^2^D^1^KL^2^I^1^XXL^2^XXY
Palm	B'	R^1^XXXXXXG^2^XP^1^Q^2,3^G^2,3^SX^3^XS^2^P^2,3^XL^1^F[NS]IYL^1^XXL^2^XE^1^XLXNXX
Palm	C	[YL]XR^1^Y^2,3^A^1,3^D^2,4^D^2,4^I[IL]V^1^X[VS]XXXKN
Palm	D	KXXLDXXTXFCXXIG^2^L^1^T^1^VN^1^XXK^2^T^1^K[IT]

Alternative amino acid residues are in brackets. 'X' is any residue. 1 – residue present in three or more of the six aligned sequences; 2 – residue present in four or more of the six aligned sequences; 3 – residue in contact with nucleic acid template, dNTP or Mg^2+ ^; 4 – point mutations of this residue abolish catalytic activity.
